# Correction: Replication Study: Inhibition of BET recruitment to chromatin as an effective treatment for MLL-fusion leukaemia

**DOI:** 10.7554/eLife.34573

**Published:** 2018-01-08

**Authors:** Xiaochuan Shan, Juan José Fung, Alan Kosaka, Gwenn Danet-Desnoyers, Reproducibility Project: Cancer Biology

Shan X, Fung JJ, Kosaka A, Danet-Desnoyers G, Reproducibility Project: Cancer Biology. 2017. Replication Study: Inhibition of BET recruitment to chromatin as an effective treatment for MLL-fusion leukaemia. *eLife* **6**:e25306. doi: 10.7554/eLife.25306.

Published 27, June 2017

This is a corrigendum for Replication Study: Inhibition of BET recruitment to chromatin as an effective treatment for MLL-fusion leukaemia, which includes the following corrections:

First, the direction of the effect size calculated for the Replication Study’s comparison of fold *BCL2* expression in K-562 cells compared to a DMSO constant of 1 (Figure 5A, top panel) is incorrect. The direction of the effect size (Cohen’s *d*) for this comparison should be *d* = −2.04 95% CI [−4.17, 0.13] (instead of the reported *d* = 2.04 95% CI [−0.13, 4.17]), which results in a change in the meta-analysis. The meta-analysis effect size should be *d* = −0.307, 95% CI [−3.57, 2.96], *p* =0.854 (instead of the reported *d* = 1.58, 95% CI [0.36, 2.79], *p*=0.011).

Second, the calculation of variance in the meta-analysis of mice treated daily with I-BET151 compared to vehicle control mice (Figure 5C) is incorrect. Subsequently, the effect size and the associated *p* value of the meta-analysis are incorrect. The effect size (Hazard Ratio) for this comparison should be HR = 3.30 95% CI [0.49, 22.18] (instead of the reported HR = 6.13 95% CI [1.11, 34.02]) resulting in a p value of *p*=0.220 (instead of the reported *p* = 0.038).

Finally, the language surrounding these comparisons in the Results and Discussion section as well as in Figure 5 and the Figure Legend (Figure 5) have been amended to account for these corrections. Additional r code for the meta-analysis can be found on the Open Science Framework: https://osf.io/vfp47/.

The corrected Figure 5 is shown here:

**Figure 1:**
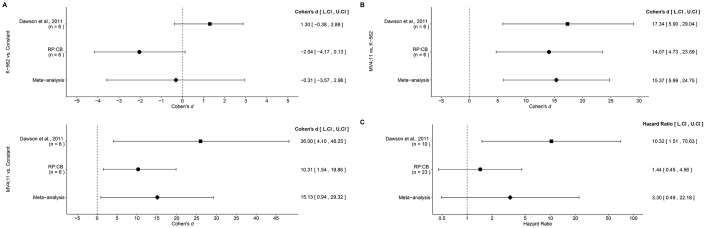


The originally published Figure 5 is also shown for reference:

**Figure 2:**
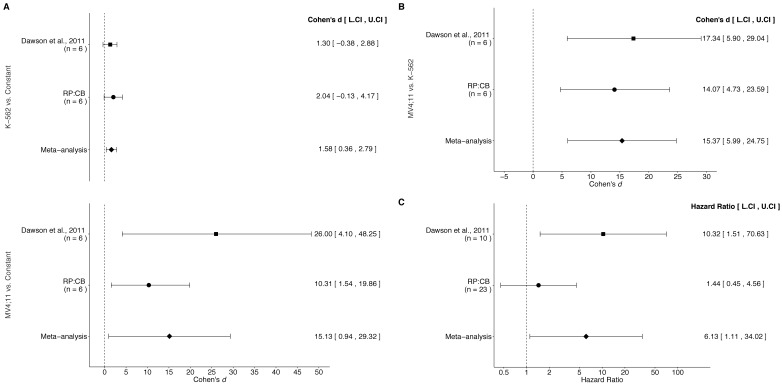


The article has now been corrected.

